# Renal Amyloidosis: Epidemiological, Clinical, and Laboratory Profile in Adults from One Nephrology Center

**DOI:** 10.1155/2022/8493479

**Published:** 2022-07-18

**Authors:** Hayet Kaaroud, Amel Harzallah, Mariem Hajji, Soumaya Chargui, Samia Barbouch, Sami Turki, Raja Trabelsi, Rim Goucha, Fatma Ben Moussa, Hedi Ben Maiz, Fethi Ben Hamida, Ezzeddine Abderrahim

**Affiliations:** ^1^Department of Medicine A, Charles Nicolle Hospital, Tunis, Tunisia; ^2^Laboratory of Renal Pathology LR00SP01, Charles Nicolle Hospital, Tunis, Tunisia; ^3^Faculty of Medicine of Tunis, University of Tunis, El Manar, Tunis, Tunisia

## Abstract

**Background:**

Renal amyloidosis is one of the main differential diagnoses of nephrotic proteinuria in adults and the elderly. The aim of this study with the most important series in our country is to contribute to the epidemiological, clinical, and etiological study of the renal amyloidosis.

**Methods:**

In a retrospective study carried out between 1975 and 2019, 310 cases of histologically proven and typed renal amyloidosis were selected for this study.

**Results:**

There were 209 men and 101 women with a mean age of 53.8 ± 15.4 years (range, 17–84 years). Of the 310 cases, 255 (82.3%) were diagnosed with AA renal amyloidosis and 55 (17.7%) with non-AA amyloidosis. Infections were the main cause of AA amyloidosis, and tuberculosis was the most frequent etiology. The period from the onset of the underlying disease to diagnosis of the renal amyloidosis was an average of 177 months. The most frequent manifestations at the time of diagnosis were nephrotic syndrome (84%), chronic renal failure (30.3%), and end-stage renal disease (37.8%). After a medium follow-up of 16 months (range, 0–68 months), mortality occurred in 60 cases.

**Conclusions:**

Given the high frequency of AA amyloidosis in our country, awareness of the proper management of infectious and chronic inflammatory diseases remains a priority in reducing the occurrence of this serious disease.

## 1. Introduction

Systemic amyloidosis can occur due to a variety of conditions, from plasma cell dyscrasia to chronic inflammatory conditions. It can also be caused by the presence of inherited amyloidosis as a mutation in a nonamyloid protein or as a mutation that affects the amyloid protein itself [1].

Despite this biochemical and etiological heterogeneity, renal amyloidosis is dominant in several forms of systemic amyloidosis [2]. It represents one of the main differential diagnoses of nephrotic proteinuria in adults.

It is necessary to establish the etiology to treat the underlying condition, especially in light chain (AL) amyloidosis, whose prognosis was significantly improved over the last decade with more effective therapy.

We aimed to determine the epidemiological, clinical manifestations, and etiology profile of renal amyloidosis with albuminuria in Tunisian adult and elderly patients.

Given the paucity of the studies in underdeveloped countries, we believe that our experience can enrich the understanding of this disease.

## 2. Patients and Methods

### 2.1. Study Design

In a retrospective study conducted in the Department of Internal Medicine A in Charles Nicolle Hospital, we reviewed the records of patients with biopsy-proven amyloidosis evaluated over a period of 44 years between 1975 and 2019.

We included all adults who were hospitalized with renal involvement and in whom amyloidosis was histologically proven and typed in our unit.

We excluded from this study the patients whose clinical data were incomplete, amyloidosis typing was not done, and those in whom amyloidosis was confirmed before hospitalization.

### 2.2. Sampling Technique

We evaluated all cases diagnosed as definite or possible amyloidosis.

Since 1997, to confirm the diagnosis of amyloidosis, we first performed salivary gland biopsy and then if negative, kidney biopsy.

The diagnosis of amyloidosis was confirmed by a positive Congo red staining examined with the apple-green birefringence under polarized light microscopy and by Cristal violet under optic microscopy.

An immunofluorescence study was performed using the polyclonal antisera against human IgG, IgM, IgA, C3, C1q, and kappa and lambda light chains.

To distinguish between the fibrils of nonamyloid A (AA) and AA amyloidosis, we used the Wright's technique [3]. The tissue sections were pretreated with potassium permanganate prior to Congo red staining. From 2000, we introduced immunohistochemical staining with anti-AA antibody DAKO.

The clinical, laboratory, and epidemiological data were recorded at the time of biopsy and were subsequently evaluated together with the histopathological findings.

Patient data were reviewed on the history of hereditary, chronic inflammatory, or infectious diseases, and, if possible, the time between the disease and the renal failure, age, gender, renal symptoms, extrarenal manifestations, chemical parameters, electrocardiogram, and echocardiography data.

When possible, the precipitating factor for the renal amyloidosis was determined from the available clinical and laboratory information. The causes of non-AA amyloidosis were not well identified due to an insufficient panel of antibodies for the immunohistochemical study and the unavailability of laser microdissection (LMD) and mass spectrometry (MS).

The course of the disease in the patients was monitored with respect to the renal function recovery, the disappearance of proteinuria, the need for dialysis support, and death.

### 2.3. Definitions

The clinical and laboratory variables were defined as follows:

- Adult patient if age >16 years and an elderly patient if age ≥65 years
- Renal involvement: albumin excretion >0.5 g in a 24-hour urine collection time
- Nephrotic syndrome: nephrotic proteinuria (≥3 g/24 h) with hypoalbuminemia (serum albumin <3 g/dl)
- The estimated glomerular filtration rate was calculated using the MDRD/ml/min/1.73m^2^ equation
- Chronic renal failure (CRF): creatinine clearance by the MDRD <60 ml/min/1.73 m^2^; end-stage renal disease (ESRD): creatinine clearance the MDRD <15 ml/min/1.73 m^2^
- Hypercholesterolemia: cholesterol level greater than 5.8 mmol/l
- Anemia: hemoglobin less than 13 g/dl in men and 12 g/dl in women
- Hyperleukocytosis: white blood cell count more than 10 × 10^3^/mm^3^
- Thrombocytosis: platelet count greater than 400 × 10^3^/mm^3^
- Hypertension: systolic blood pressure (BP) ≥140 mm·Hg and/or diastolic BP ≥90 mm·Hg
- Hypotension: systolic BP <110 mm·Hg and/or diastolic BP <70 mm·Hg
- Orthostatic hypotension: a decrease in the systolic blood pressure of at least 20 mm·Hg and/or diastolic blood pressure of at least 10 mm·Hg after 3 minutes of orthostatic position
- Cachexia: body mass index less than 18.5 kg/m^2^
- Complete remission: albuminuria less than 0.3 g/24 h with normal renal function
- Partial remission: decrease of albuminuria less than 2 g/24 h and/or improvement in renal function

### 2.4. Statistical Analysis

Statistical analysis was performed with the help of StatView software. A descriptive analysis was used to study the continuous variables (means, medians, standard deviation, and ranges) and the frequencies of categorical variables (N and percentages). The means were compared using the Student's *t*-test or, when appropriate, the Mann–Whitney nonparametric test. Percentages were compared with the Pearson's hi-square test, or, when appropriate, Fisher's exact test. *P* < 0.05 was considered to denote statistical significance.

## 3. Results

**Figure 1:**
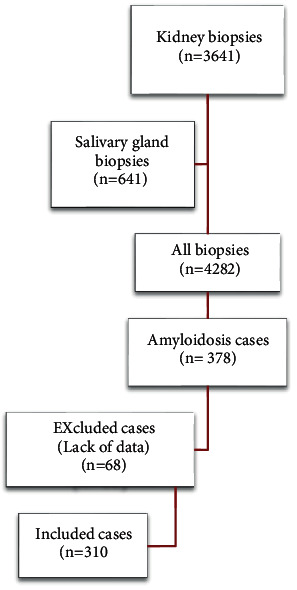
Flow chart of the study design.

**Figure 2:**
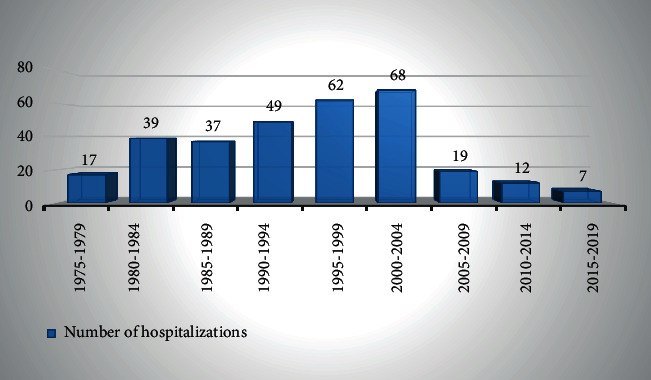
Number of hospitalization time.

During the 44-year period, 3641 nontransplant kidney biopsies and 641 salivary gland biopsies were performed. Amyloidosis was identified in 378 cases. According to our inclusion criteria, 310 cases were included (Figure 1). The number of admissions according to the different 5-year period is shown in Figure 2.

The clinical profile of the patients is summarized in Table 1.

There were 209 men and 101 women with a gender ratio of 2.06.

The mean age of the patients was 53.8 ± 15.4 years (range, 17–84 years). The average age of men was 53.8 years, and it is the same for women. The AA amyloidosis patients were younger (median age 52.8 years) compared to those with non-AA amyloidosis (median age 58.5 years) (Table 1). The most affected age group was 17 to 64 years (74%), followed by 65 (26%). Amyloidosis was type AA in 100% of the cases for age below 30 years, in 88% of the cases for an age range between 30 and 50 years, and in 76.7% for age above 50 years.

In 117 patients (37.7%), a precipitating factor for renal amyloidosis was identified from the clinical records, that is, infection in 96 cases (31%) (pulmonary in 72 cases (23%), urinary in 16 cases (5.2%), other infections in 8 cases (2.6%)) and surgery in 21 cases (6.8%).

Edemas were present at the time of diagnosis in 83.7% of the cases.

One hundred and twenty-three (39.7%) patients had hypotension, and 56 (18.1%) patients had hypertension. For hypertensive patients, the CRF has been reported in 51 cases (16.4%), including 30 cases (9.7%) of ESRD.

The organs most affected, other than the kidneys, in our patients were the digestive tract (55.4%) manifested by chronic diarrhea, malabsorption, and bleeding. The second organ affected was the soft tissue (38.3%), including macroglossia and periorbital ecchymosis. This location was more frequent in AA amyloidosis than in non-AA amyloidosis.

Hepatomegaly and/or cholestasis were noted in 18% of the cases, while the frequency of splenomegaly was 6.8% (Table 1). Of the 17 patients with thyroid goiter, 4 had hypothyroidism related to confirmed histological amyloidosis in 2 cases, and one patient had hyperthyroidism. Cachexia was observed in 41 cases (13.2%).

The median serum creatinine at the baseline was 408 *μ*mol/l (range, 37–3294 *μ*mol/l). Initially, normal renal function was detected in 99 patients (31.9%), CRF was present in 211 patients (68%), and among them, 117 patients (37.7%) had ESRD that did not require dialysis. Renal replacement therapy was started in 68 patients (21.9%) at the time of diagnosis.

The median protein excretion was 6.4 g per day (range, 1–26). The median serum albumin level was 17 g/l (range, 4–42); serum albumin less than 20 g/L was observed in 61% of cases.

The serum albumin was significantly lower in cases with AA amyloidosis (17 vs. 21.3 g/L, *P*=0.0003), and the median serum creatinine as well as the magnitude of proteinuria was higher in AA compared to non-AA amyloidosis (serum creatinine, 427.4 vs. 230 *μ*mol/L; proteinuria, 6.5 vs. 6 g/24 h) but failed to attain statistical significance.

Nephrotic syndrome was present in 84% of the patients and was associated with chronic renal failure in 56% of the cases.

The median cholesterol level was 6.68 mmol/l (range, 2.1–26) with hypercholesterolemia in 64% of the patients.

Anemia occurred in 80% of patients (80.6% for women and 79% for men) with an average hemoglobin level at 10.3 g/dl (range, 4–17.6 g/dl). The frequency of anemia unrelated to renal failure has been noted in 21% of cases.

The frequency for hyperleukocytosis was 35.5% and 41.6% for thrombocytosis.

Hematuria not related to urinary tract infection was noted in 15.6% and glucosuria in 40% of cases.

The electrocardiogram performed in 181 patients (58.4%) showed the typical pseudoinfarct pattern in 15.4%, low voltage in 63.6%, arrhythmia in 10.3%, conduction disorder in 9.8%, and repolarization disorders in 33.9%.

On the chest radiograph performed in 248 cases (80%), there was pleural effusion in 29% of the cases, residual signs of tuberculosis in 30.7% of the cases, emphysema in 11% of the cases, and an infection focus in 18.6% of the cases. Cardiomegaly was present in 27.7% of the cases.

Echocardiography data obtained from 98 patients (31.6%) showed abnormalities in 64% of them.

Renal echography highlighted normal kidneys in 61% of the cases, kidney hypertrophy in 26.4% of the cases, and atrophic kidneys in 12.6% of the cases.

In the first intention, biopsy was performed with the kidney needle in 204 cases (65.8%) and with the accessory salivary glands in 100 cases (32.3%). The renal histological examination was performed at autopsy in 6 cases (1.9%).

In renal biopsy, the predominant sites of amyloid deposition were glomeruli (99.4%) and vascular (99.4%), followed by tubulointerstitial deposits in 54% of cases.

Amyloidosis typing was performed using the Wright's technique in 62 cases (20%), immunohistochemistry in 244 cases (78.7%), and by both methods in 4 cases (1.3%).

The cases were classified as AA type (255 cases, 82.3%) and non-AA type (55 cases, 17.7%).

Amyloid deposits have been detected in other organs, in symptomatic patients, during surgery or at autopsy (tongue: 1 case; temporal artery: 1 case; digestive tract: 5 cases; liver: 1 case; peritoneum: 1 case; adrenals: 2 cases; synovial: 1 case; thyroid: 2 cases; lymphadenopathy: 1 case).

The most frequent underlying disorder of AA amyloidosis was chronic infection noted in 149 cases (48.1%) and especially tuberculosis (27.7%). For other causes, we observed chronic inflammatory diseases in 28 cases (9%), periodic fever syndrome in 19 cases (6.2%), and neoplasia in 5 cases (1.6%) (Table 2).

Of the 55 patients with non-AA amyloidosis, 15 (4.8%) had multiple myeloma (Table 2).

The etiology was undetermined in 54 cases (17.4%) of AA amyloidosis and in 36 cases (11.6%) of non-AA amyloidosis.

The median time between the occurrence of the first symptoms of the underlying disease and the diagnosis of amyloidosis has been defined in only 122 cases (39.4%). It was variable, ranging from 0 to 604 months with a median of 177 months. This delay was 182 months for type AA and 18.5 months for type non-AA.

Complications were dominated by infection and thrombosis was observed, respectively, in 30% and 8% of the cases.

Symptomatic treatment was conducted in all the cases. Colchicine was prescribed in 47 cases (15.2%). Specific treatment to the etiology was performed when the cause was identified.

One hundred and fifty patients were lost to follow-up on discharge (48,4%). Among the remaining 160 patients (51.6%), 60 (37.5%) died (20 non-AA, 40 AA), 34 of them (56.7%) during hospitalization, and 26 (43.3%) after an average follow-up of 16 months (2–68 months). Complications such as sepsis (32%) and CRF (54%) were the leading causes of death. In only 8% of cases, the death was related to other causes.

Of the 100 remaining patients (32.3%), after an average follow-up of 27 months (2–228 months), 44 (14.2%) had ESRD, 23 (7.4%) of whom required hemodialysis, 26 (8.4%) had CRF, 27 (8.7%) had normal renal function, 1 (0.3%) developed complete remission, and 2 (0.6%) developed partial remission.

## 4. Discussion

Renal amyloidosis remains a significant disease in our country, much higher than that reported in several studies (0.65–4%) [4–7]. However, there has been a decrease in the number of cases since 2004, which is due to the gradual opening of other nephrology units in our country [8].

In the present study, the median age at diagnosis was around 54 years, close to that of underdeveloped countries but less than the developed ones. A quarter of our patients were over 65 years old [5–7, 9].

AA amyloidosis has been reported to be the most common form of amyloidosis in children and young adults [9]. In this study, amyloidosis was type AA in all patients aged 30 years or less; the percentage decreased to 76.7% after 50 years.

Our patients with non-AA amyloidosis were significantly older (58.5 years) than those with AA amyloidosis (52.8 years), which is consistent with the data from the literature [5, 6, 9–11].

In terms of gender, men were more affected than women. The distribution was similar for some authors [12–15]. However, according to others, women exhibited a similar or predominant predisposition [10, 16].

Our results showed that the gender was prevalent in both the cases of non-AA (61.8%) and AA (68.6%). Some previous studies reported similar conclusions, while others showed different ones in which women were more affected by AL amyloidosis [6, 11].

The higher prevalence of AA amyloidosis in men in this study was partly due to a higher frequency of chronic bronchitis related to increased smoking.

In our cohort, AA amyloidosis was more common than non-AA amyloidosis (82.3% VS 17.7%). This difference can be partially explained by a selection bias consisting in including patients mainly from the lower socio-economic class.

In our patients with renal AA amyloidosis, the latency period between the onset of the inflammation and the first clinical signs was 182 months, which is comparable to that described in the literature [5].

The deposition of AA amyloid fibrils is the result of a prolonged inflammatory state [17–21].

In some developed countries, the serum amyloid A (SAA) levels can be monitored and used to guide response to the treatment [21, 22].

Additional genetic and environmental factors are involved in the susceptibility to the occurrence of amyloidosis. Among the two genes encoding SAA proteins, the *SAA1* locus plays a role in amyloidosis sensitivity [23, 24]. Neither quantification of SAA levels nor the sequencing of the *SAA1* locus are common practices in our experience.

In this study, infections were the main cause of AA amyloidosis, and tuberculosis was the most common etiology. Other studies have also described tuberculosis as the most common etiology of renal amyloidosis [9]. The other types of infection described in our patients are similar to those reported in the different series [5, 9, 22, 25, 26]. However, we had a high unusual frequency of echinococcosis.

The second cause was chronic inflammatory disease dominated by rheumatic diseases and mainly chronic polyarthritis whose frequency remains high due to a lack of new effective drugs.

Hereditary auto-inflammatory syndrome, which is associated with various periodic fevers, of which familial Mediterranean fever is the best known, is a nonnegligible cause since consanguineous marriages are still frequent in our population.

Patients with familial Mediterranean fever who are homozygous for the M694V mutation are at increased risk for the development of amyloidosis [27]. The large number of hereditary auto-inflammatory syndromes among our cases can be explained in part by the frequency of this mutation in our population [28].

As described previously, AA amyloidosis has also been associated with Behcet's disease, inflammatory bowel disease, chronic inflammatory diseases, and malignancies [7, 14, 22, 29, 30].

We have a higher frequency of AA amyloidosis with no disease identified (17.4%) than previously reported at 6% to 9.9% [10, 22]. This is certainly due to an underdiagnosis, mainly of hereditary forms, linked to an innate error of inflammatory response in the immune system.

Castleman's disease deserves to be investigated in the presence of AA amyloidosis without an obvious cause [31].

Regarding etiology, our results contrast sharply with studies from the developed countries where amyloidosis was type AA—only 4.8% to 40%—and the chronic inflammatory diseases were the most common cause [10, 22, 25, 32].

AL amyloidosis is more prevalent in the developed countries with a reported incidence of approximately 12 cases per million persons per year [1, 11, 14, 33].

Among the 55 cases of non-AA amyloidosis, a condition that may be related to amyloidosis was identified only in 19 cases.

The diagnosis of the causes for non-AA amyloidosis was unreliable in this study. The frequency of myeloma was certainly underestimated, especially in old files because the diagnostic criteria have continued to change and there are certain cases of transthyretin amyloidosis that were underdiagnosed due to lack of MS or genetic study.

The biggest challenge that has emerged in recent years is the detection and correct diagnosis of the hereditary amyloidosis and its differentiation from AL [1, 12, 34–38].

The ATTR amyloidosis derived from a transthyretin variant is the most common hereditary amyloidosis in the world [12, 37].

The frequency of extrarenal manifestations in our patients does not quite agree with the recent studies from the developed countries who use radiolabeling serum amyloid protein 123 I (123 I-SAPS), a sensitive method for detecting the visceral amyloid deposits, even in tissues not considered clinically involved [21, 22, 39]. The evaluation of cardiac involvement is largely underestimated in our study because biomarkers that include cardiac troponin, N-terminal probrain natriuretic protein (NT-pro-BNP), and magnetic resonance imaging are not common practice in our hospital [40–43].

The clinical presentation of renal involvement is generally the same regardless of the amyloid type [20, 44]. There is often a precipitating factor for amyloid nephropathy. The pulmonary infection was the main precipitating factor in our patients; however, renal amyloidosis was revealed by surgery in 6.8% of the cases.

The present patients had an average of 24-hour urinary protein excretion of 6.4 g per day (range, 1 to 26 g/day). Only 16% of our patients had nonnephrotic proteinuria suggesting weak glomerular involvement. In our cases, albuminemia was lower than 20 g/l in 61% of cases with cachexia in 13.2%.

The nephrotic syndrome, described in 64 to 86%, was detected in 84% of our patients and was complicated with venous thrombosis in 8% of the cases [7, 11, 16, 44].

Impaired renal function in our patient was observed in 68.1%, which is consistent with other studies [6, 7, 9]. ESRD, described in 5 to 32.3% of cases [5, 16], was noted in 37.8% in our series.

Hypertension, which is an uncommon feature of amyloidosis present in 7.3 to 12.8% in some series, was observed in 17.3% of our patients [9, 11, 16].

Hematuria is considered a rare consequence of renal amyloidosis, and its frequency in our patients was 15.6% [7]. This may reflect the severity of damage caused by intravascular and urinary amyloid deposits or thrombosis of the renal veins.

The anemia was often related with renal failure in 79% of the patients, and in 21%, it was the consequence of an inflammatory state, a digestive bleeding, or hematological disorder of the myeloma.

Currently, tissue biopsy remains the gold standard for the diagnosis of amyloidosis, despite advances in noninvasive imaging [2, 21, 45, 46].

The diagnosis requires the histological demonstration of the amyloid deposits, typically by their pathognomonic green birefringence under polarized light upon Congo red staining [2, 47].

In renal biopsy, amyloid, especially accumulated in the glomeruli, consistently results in proteinuria, nephrotic syndrome, and the progressive development of renal insufficiency [48, 49].

The high frequency of glomerular involvement (99.4%) seen in our patient was the result of the selection criteria for renal manifestations.

The glomerular involvement was associated with vascular and/or tubulointerstitial involvement in, respectively, 99.4 and 54% of our biopsies. Normoglycemic glucosuria noted in 40% is suggestive of tubule interstitial involvement.

In patients diagnosed with systemic amyloidosis, the treatment options focus on recognizing one of the three main categories of systemic amyloidosis: AL, AA, and the ever-expanding group of hereditary amyloidosis.

The potassium permanganate technique is used as the initial method for typing amyloidosis, and it should no longer be practiced [3, 44]. Immunofluorescence and immunohistochemistry with custom antibodies remain the most used methods to diagnose and type amyloid worldwide [2, 37, 44].

The present study has some limitations because we do not have the full panel of antibodies; therefore, on our own sample, we have classified renal amyloidosis in AA and not AA.

We certainly overdiagnosed some nonreactive cases such as AL because the immunofluorescence is a questionable method.

Currently, the gold standard methods for typing amyloidosis are LMD and MS with a sensitivity of almost 100%.

Genetic testing should always be complementary to other diagnostic techniques [44].

The prognosis for renal amyloidosis was poor in our patients due to a high mortality rate. As described in previous reports, the causes of death were dominated by infections and ESRD [6, 7, 11, 22, 26].

However, with early diagnosis and treatment, increased reports of remission and prolonged survival were reported [14]. The remission was rare in our experience since we noted only three cases including one complete remission and two partial remissions.

## 5. Conclusions

In this study, the renal amyloidosis affected the young adult males. Many patients were still diagnosed with already established advanced organ damage due to the lack of routine screening for albuminuria. Proteinuria with or without nephrotic syndrome associated with chronic renal failure was the most common clinical finding. The renal AA amyloidosis was predominant, and its main cause was tuberculosis.

Due to an insufficient antibody panel and the unavailability of the LMD/MS, AL amyloidosis was not well identified, and the hereditary amyloidosis remains undiagnosed.

Mortality was high due to late diagnosis and rapid progression to chronic renal failure.

The prevention of AA amyloidosis is based on screening the risk patients at a presymptomatic stage, the eradication of infectious diseases, and the treatment of the underlying etiology; the frequency of this condition can decrease dramatically, especially since other types of amyloidosis were not frequent in our patients.

## Figures and Tables

**Table 1:** Clinical and histological data of 310 patients with renal amyloidosis.

Parameter	Value	AA amyloidosis (*n* = 255)	Non-AA amyloidosis (*n* = 55)	*P* value
Male gender	2.06 (209 M, 101 F)	2.18 (175 M, 80 F)	1.6 (34 M, 21 F)	NS
Age (years)	53.8 ± 15	52.8	58.5	0.012
Organ's involvement, *n* (%)				
Digestive tract	61/110 (55.4%)	51	10	NS
Soft tissues	87/227 (38.3%)	63	24	0.004
Liver	50/277 (18%)	42	8	NS
Spleen	18/264 (6.8%)	13	5	NS
Lymphadenopathy	21/168 (12.5%)	15	6	NS
Hypoacusis	29/122 (23.8%)	26	3	NS
Goiter	17/69 (24.6%)	13	4	NS
Peripheral neuropathy	3/14 (21.4%)	0	3	NS
Clinical signs, *n* (%)				
Edema	241/288 (83.7%)	200	42	NS
Arterial hypotension	123/310 (39.7%)	101	22	NS
Orthostatic arterial hypotension	50/310 (16.1%)	37	13	NS
Arterial hypertension	56/310 (18.1%)	45	11	NS
Biology				
Proteinuria (g/24 h)	6.4 (1–26)	6.5	6	NS
Serum albumin (g/l)	17 (4–42)	17	21.3	0.0003
Nephrotic syndrome, *n* (%)	84%	212	39	NS
Creatinine (*μ*mol/l)	408 (37–3294)	427.4	230	0.04
Normal renal function, *n* (%)	99 (31.9%)	79	20	NS
Chronic renal failure, *n* (%)	94 (30.3%)	75	19	NS
ESRD, *n* (%)	117 (37.8%) (68 required HD)	101	16	NS

HD: hemodialysis; ESRD: end-stage renal disease; F: female; M: male; NS: not significant.

**Table 2:** Underlying disease of renal amyloidosis

Underlying disease with AA amyloidosis	Number (*n* = 255)	%
Chronic sepsis	149	48.1
Tuberculosis	86	27.7
Bronchiectasis	48	15.5
Osteomyelitis	7	2.3
Echinococcosis	5	1.6
Others	3	1
Chronic inflammatory disease	28	9
Rheumatoid arthritis	12	3.9
Ankylosing spondylitis	8	2.6
Juvenile idiopathic arthritis	1	0.3
Crohn's disease	3	1
Ulcerative colitis	1	0.3
Behcet's disease	1	0.3
Takayasu	1	0.3
Scleroderma	1	0.3
Periodic fever syndromes	19	6.2
Neoplasia	5	1.6
Cancer solid (bladder: 1 case, colon: 1, lung: 1)	3	1
Lymphoma (Hodgkin: 1, breast: 1)	2	0.6
Unknown	54	17.4
Underlying disease with non-AA amyloidosis	(*n* **=** **55)**	
Multiple myeloma	15	4.8
Gammopathy	3	1
Waldenstrom's syndrome	1	0.3
Unknown	36	11.6
Total	**310**	**100**

## Data Availability

The datasets generated during the current study are available from the corresponding author on reasonable request.

## Abbreviations

AL: Light-chain amyloidosis
AA: Amyloid A
LMD: Laser microdissection
MS: Mass spectrometry
SAA: Serum-amyloid A
CRF: Chronic renal failure
ESRD: End-stage renal disease
BP: Blood pressure
ATTR: Transthyretin.
